# Reducing indoor virus transmission using air purifiers

**DOI:** 10.1063/5.0064115

**Published:** 2021-10-01

**Authors:** Talib Dbouk, Frederic Roger, Dimitris Drikakis

**Affiliations:** 1 University of Nicosia, Nicosia CY-2417, Cyprus; 2 Institut Mines-Telecom, Paris, France; 3 Institut Mines-Telecom Nord Europe, University of Lille, Lille, France; 4 Univ. Lille, ULR 7512—Unité de Mécanique de Lille—Joseph Boussinesq (UML), F-59000 Lille, France

## Abstract

Air purifiers are limited to small polluting airborne particles and poor air circulation (fan) for bringing airborne particles inside the device. Thus, the optimal utility of domestic air purifiers (DAPs) for eliminating airborne viruses is still ambiguous. This paper addresses the above limitations using computational fluid dynamics modeling and simulations to investigate the optimal local design of a DAP in an indoor space. We also investigate the integrated fan system and the local transport of airborne viruses. Three different scenarios of using standard DAP equipment ( $144\;\;m^{3}/h$) are explored in an indoor space comprising a furnished living room $6\times 6\times 2.5\;\;m^{3}$. We show that the local positioning of a purifier indoors and the fan system embedded inside it can significantly alter the indoor airborne virus transmission risk. Finally, we propose a new indoor air circulation system that better ensures indoor airborne viruses' local orientation more efficiently than a fan embedded in a standard DAP.

## I. INTRODUCTION

The COVID-19 pandemic has motivated research to understand environmental conditions of airborne virus transmission. Experimental approaches are still limited in accurately measuring or quantifying airborne virus particles suspended in the air.^1^ Computational modeling provides an alternative approach in simulating airborne virus transmissions. The above was evidenced through several recent studies since the start of the pandemic.^2–13^ The studies by Dbouk and Drikakis^2–6^ also led to an epidemiology forecast physics-based model that considers weather seasonality^14^ and corrections to pandemic data.^15^ Before the COVID-19 pandemic, research studies were also published,^16,17^ but there is a significant growth of published papers and increasing motivation to address scientific and technical issues since the start of 2020.

The risk of airborne virus transmission indoors is higher than outdoors.^18^ Furthermore, face masks do not stop virus particles’ emissions. They only reduce them and orientate their direction.^3^ Thus, there is a need to develop technologies for reducing the indoor transmission of viruses. Research in understanding the optimal location of indoor air purifiers is scarce.^19^

The present study aims to advance knowledge regarding deactivating virus particles inside a purifier device and investigate the air circulation system absorbing the particles. We employed computational fluid dynamics modeling and simulation to address the following questions: (a) What is the optimal local design of a domestic air purifier (DAP) inside an indoor space (e.g., a living room)? (b) What is the effect of the air circulation system on the transport of airborne viruses? (c) Does the emerging DAP technology provide sustainable indoor air quality in deactivating airborne viruses as small as 140 nm?

**FIG. 1.**
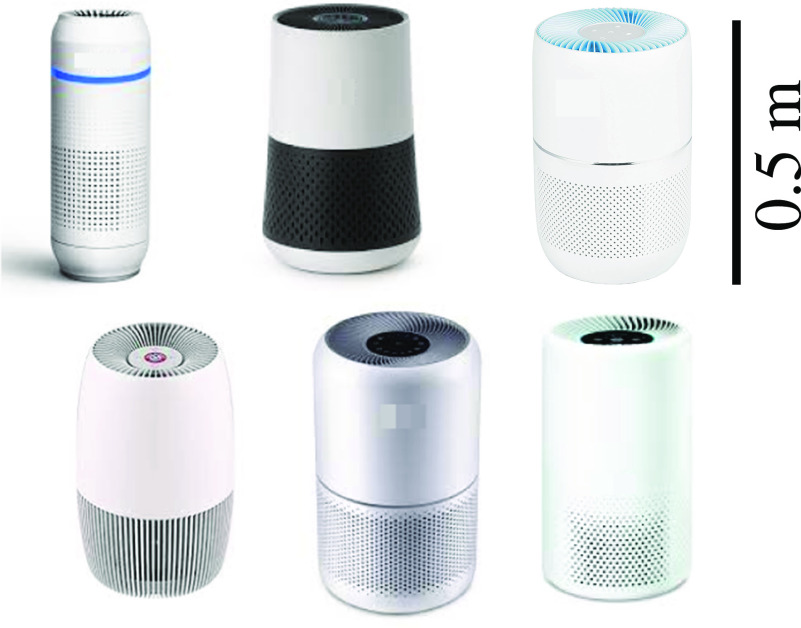
An example of different cylindrical designs of emerging domestic indoor air purifiers devices.

We focus on emerging cylindrical designs of indoor air purifiers (see Fig. 1). We investigated three different scenarios using a standard DAP equipment ( $144\;\;m^{3}/h$) in an indoor space represented by a furnished living room ( $6\times 6\times 2.5\;m^{3}$). First, we injected a stratified layer of 1024 airborne virions, uniformly distributed along the room cross section, 1.7 m above the ground level. This layer mimics virus particles inside a room, and an air purifier device should rapidly clean that. Finally, we propose a high-speed innovative air purification concept that can rapidly and continuously eliminate airborne viruses under the continuous presence of infected individuals indoors.

## II. COMPUTATIONAL METHOD

### A. Scenarios and computational domain

**FIG. 2.**
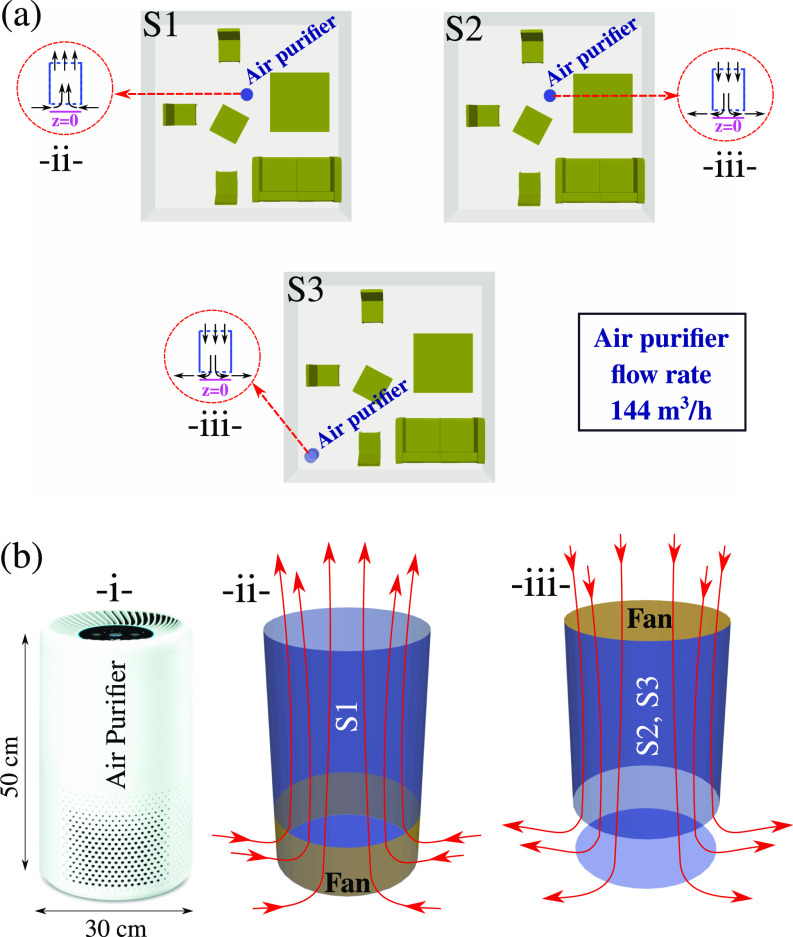
(a) Three different scenarios of using a domestic air purifier device operating at $144\;m^{3}/h$ inside a furnished living room. S1: purifier placed in the middle of the room with the air suction mechanism at the bottom side of the purifier device; S2: purifier placed in the middle of the room with the air suction mechanism on the top; S3: purifier placed in the corner of the room with the air suction mechanism on the top. (b) A real example of the purifier device (i) with different systems of fan integration (ii and iii).

Three different scenarios are investigated (Fig. 2) for various local positioning of a cylindrical design of a DAP inside a furnished living room ( $6\times 6\times 2.5\;m^{3}$) aiming to mimic real DAP devices (i.e., see Fig. 1). The scenarios S1, S2, and S3 are shown in Fig. 2. We also investigated two different positions of the purifier in the room: center and corner.

**FIG. 3.**
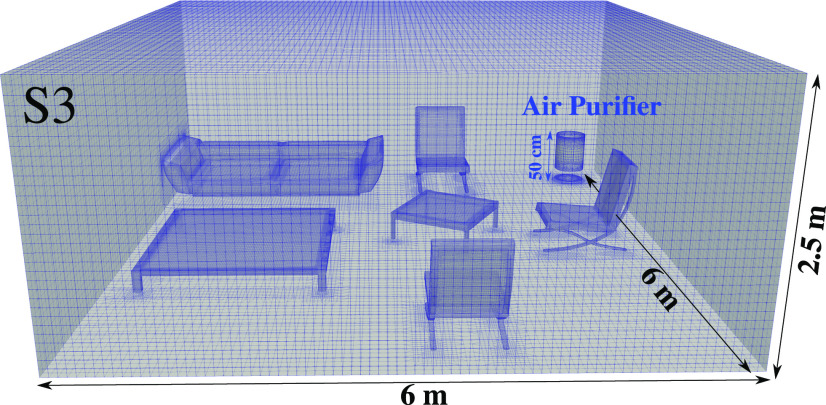
An example of the three-dimensional computational domain showing the local hexahedral mesh. Mesh refinement is applied close to the furniture surfaces and inside the air purifier device of height h=50 cm and diameter D=30 cm placed at the corner (scenario S3 of Fig. 2) inside a furnished living room of $6\times 6\times 2.5\;m^{3}$.

The computational domain for S3 is shown in Fig. 3 including the room and the air purifier (50 cm height and 30 cm in diameter operating at $144\;m^{3}/h$). Local mesh refinement is applied near the furniture surfaces to enhance the accuracy and better capture the dynamics of the local particles near these surfaces. To mimic the presence of virus particles inside the room, we injected virions through a uniformly distributed stratified layer of 1024 spherical nanoparticles of diameter $D_{p}=140\;\text{nm}$. This layer is set at 1.7 m above the ground, which corresponds to an average height of a human mouth. This is like a worst-case scenario where several local persons may expel virus particles at different local positions inside the room. They should be cleaned by the air purifier device as fast as possible. The emission process can be continuous and cyclic, with many more virus particles suspended in the indoor air. We focus on the minimum period needed to remove virion particles from the 1024 particles.

### B. Indoor air dynamics

To study the indoor air dynamics, we use the three dimensional (3D) compressible Navier–Stokes equations that include the conservation of mass, momentum, and energy,

$$\frac{\partial \rho_{f}}{\partial t}+\vec{\nabla }\cdot (\rho_{f}\vec{U_{f}})=0,$$ (1)

$$\frac{\partial (\rho_{f}\vec{U_{f}})}{\partial t}+\vec{\nabla }\cdot [\rho_{f}\bar{\bar{U_{f}⊗U_{f}}}]=-\vec{\nabla }P+\vec{\nabla }\cdot \bar{\bar{\tau }},$$ (2)

$$\frac{\partial (\rho_{f}E)}{\partial t}+\vec{\nabla }\cdot ((\rho_{f}E+P)\vec{U_{f}})=\vec{\nabla }\cdot (\bar{\bar{\tau }}\cdot \vec{U_{f}}).$$ (3)

*ρ_f_*, ⊗, $\vec{U_{f}}$, *P*, *E*, and $\bar{\bar{\tau }}$ are the indoor air density, tensorial product operator, the air velocity field vector, the pressure, the internal energy, and the shear stress tensor, respectively.

### C. Airborne virus particles dynamics

We employ the Euler–Lagrange technique to solve the coupling between indoor air and indoor airborne virus particle dynamics. The coronavirus particles are simulated as virions (capsids) of 140 nm diameter. Each virion particle is tracked individually throughout the indoor space. We assume that the virion particles suspended in the indoor air are not subject to evaporation, which is the worst-case scenario.

The evolution of the virion particles velocity is computed by applying Newton's second law of motion such that

$$m_{p}\frac{d\vec{U_{p}}}{dt}=\sum \vec{F_{p}}(\vec{U_{p}},\vec{U_{f}},\vec{B}).$$ (4)

$\vec{U_{p}}$ is the virion velocity vector and *F_p_* are the forces acting on the virion particle (as a function of the virion velocity $\vec{U_{p}}$ and the indoor air velocity vector $\vec{U_{f}}$ interpolated at the virion particle position). $\vec{B}$ represents the external force of gravity.

The evolution of the virion particle temperature is obtained throughout the energy equation that is based on the enthalpy difference *H_p_*,

$$\frac{dH_{p}}{dt}=A_{p}\left({\dot{q}}_{\mathrm{conv}.}+{\dot{q}}_{\mathrm{abs}.}-{\dot{q}}_{\mathrm{emm}.}\right),$$ (5)

where *A_p_* is the virion particle surface area. $q_{\mathrm{conv}.}$ represents heat transfer due to convection with air. Radiative heat transfer is split in $q_{\mathrm{abs}.}$, which is gained from the environment, and $q_{\mathrm{emm}.}$, which is emitted and lost from the virion surface. The above enthalpy equation can be written as a function of the virion particle temperature *T_p_* such that

$$\frac{dH_{p}}{dt}=m_{p}c_{p}\frac{dT_{p}}{dt},$$ (6)

where *c_p_* is the virion particle specific heat capacity. For more details about the mathematical derivations of the numerical models, the readers may refer to Refs. 2–5.

### D. Initial and boundary conditions

We applied an immersed fan interface condition to mimic the purifier ventilator inside the domain [Figs. 2(b-ii) and 2(b-iii)]. The fan operates at a flow rate of $144\;m^{3}/h$, under three different scenarios S1, S2, and S3 [Figs. 2(a) and 2(b)]. The external envelope of the purifier is maintained at $T_{\mathrm{purifier}}=30\;{}^{\circ}C$, while the living room walls and the interior air are initially at $T_{w}=T_{f}=22\;{}^{\circ}C$. The internal furniture is considered to be initially in mutual thermal equilibrium with the surrounding air with $T_{\mathrm{furn}}=T_{f}=22\;{}^{\circ}C$. An embedded particles-attracter or sticking filter condition is applied inside the air purifier. This is to quantify with time how many virus particles are trapped inside the air purifier compared to other particles that stay suspended in the indoor air circulating outside the air purifier device. Particles stick (no-slip) boundary condition is applied on all the walls and the furniture surfaces. This makes it easier to differentiate between virus particles attached to the surfaces and virus particles suspended in the indoor air space (e.g., more dangerous airborne virus transmission).

**FIG. 4.**
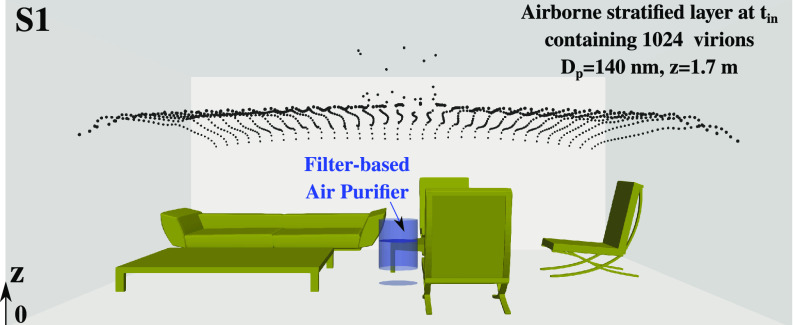
An example of scenario S1 of Fig. 2 illustrating the layer of 1024 airborne virus particles ( $D_{p}=140\;\text{nm}$) uniformly distributed at $t=t_{in}+\Delta t$ and at z=1.7 m. At the first time step Δt, we can observe that the virus particles, directly above the air purifier, are pushed upward due to the embedded fan blowing air upward as shown in Fig. 2(b) for the scenario S1 [Fig. 2(b-ii)].

We conducted 3D transient simulations, but first, we solve for an indoor airflow stabilization over some time $T\leq t_{in}$, such that *t_in_* representing the maximum time required to reach a steady state. At the instant $t=t_{in}$, we inject into the domain a horizontally ( z=1.7 m) suspended layer of 1024 airborne virus particles to be cleaned by the indoor air purifier ( h=50 cm, D=30 cm). At $t=t_{in}$, the virions layer is injected as it can be observed in Fig. 4. For $t>t_{in}$, the virus particles move inside the living space and are tracked over a maximum physical time $t_{\text{max}}-t_{in}=2.5\;\text{min}$. The virus particles are modeled as coronavirus virions (or capsids) with diameter $D_{p}=140\;\text{nm}$. The uniform local distribution of the virions in the room at $t=t_{in}$ represents the worst-case scenario, i.e., virus particles are present throughout the room.

### E. Numerical solvers

The open-source CFD code “OpenFOAM”^20^ was employed to solve the partial differential equations using the finite volume method.^21^ The Eulerian–Lagrangian framework is applied to track the airborne viruses inside the indoor space with the computational domain shown in Fig. 3. For air, we use the ideal gas law, and the air transport is represented by Sutherland's law^22^ to account for the viscosity change based on the kinetic theory of gases, e.g., appropriate for non-reacting gases. Second-order accurate numerical schemes in both time and space are used. The Lagrangian equations of the virion particles are discretized using a semi-implicit, second-order numerical method.

The pressure-velocity coupling is solved by employing the compressible PIMPLE algorithm. This algorithm is a combination of PISO (Pressure Implicit with Splitting of Operator) and SIMPLE (Semi-Implicit Method for Pressure-Linked Equations) algorithms intended for transient cases.^23^ To account for turbulence local phenomena, we solve turbulent airflow viscosity in the turbulence regime by employing the unsteady RANS technique with a k−ω−SST turbulence model.^24,25^ Mesh sensitivity analysis has been conducted according to the guidelines of Ref. 26 to adopt an accurate mesh size for the different simulations. The adopted mesh was about 2 × 10^6^ hexahedral cells with local mesh refinement near the furniture surfaces. The mesh generation method is based on four successive steps using hexahedral cells topology: (i) create primary mesh; (ii) refine the mesh; (iii) adjust the mesh to fit the main geometry; and (iv) add boundary layers near the requested patches. The total computation time of every single simulation to complete a physical time of 2.5 min was of the order of 7 days. This was accomplished employing a high-performance computing cluster (HPC), where each simulation (or scenario) was run in parallel on 256 AMD processors, each 2.25 GHz and employing 256 GB of DD-RAM.

## III. SINGLE INDOOR AIR PURIFIER DEVICE: RESULTS AND DISCUSSION

**FIG. 5.**
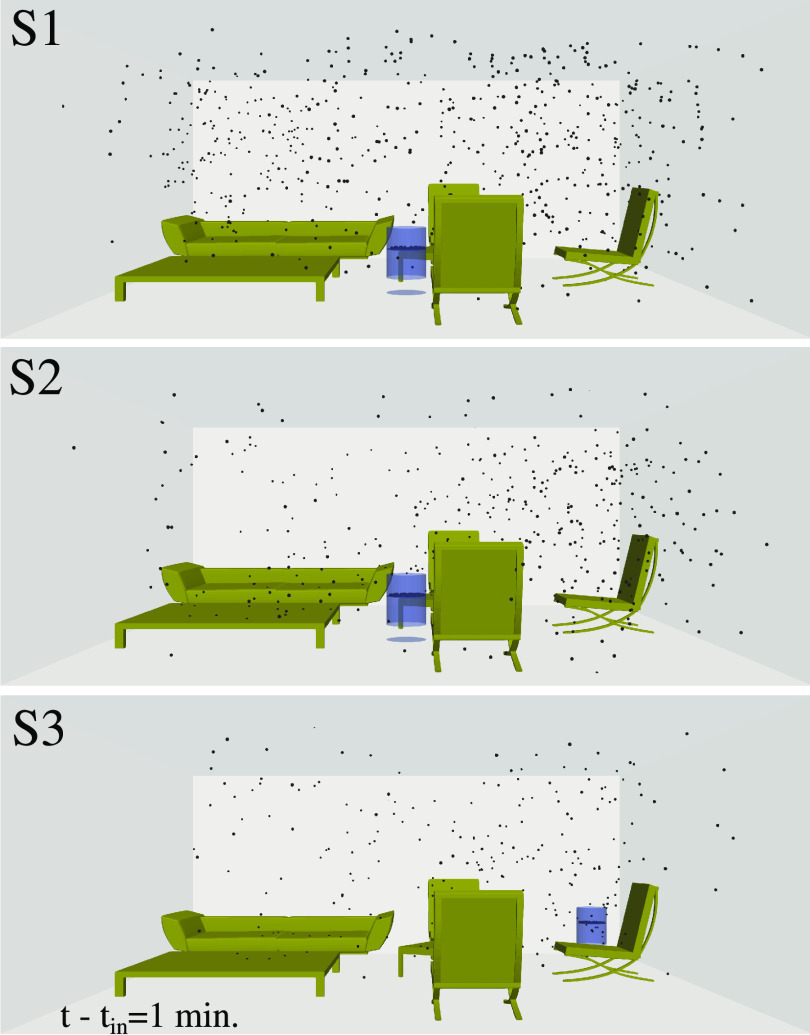
The local distribution of the airborne virus particles at $t-t_{in}=1\;\text{min}$ under the three different scenarios S1, S2, and S3 of Fig. 2. A corner position for the air purifier gives the best results, particularly in scenario S3. Multimedia views: https://doi.org/10.1063/5.0064115.1
10.1063/5.0064115.1; https://doi.org/10.1063/5.0064115.2
10.1063/5.0064115.2; https://doi.org/10.1063/5.0064115.3
10.1063/5.0064115.3

**FIG. 6.**
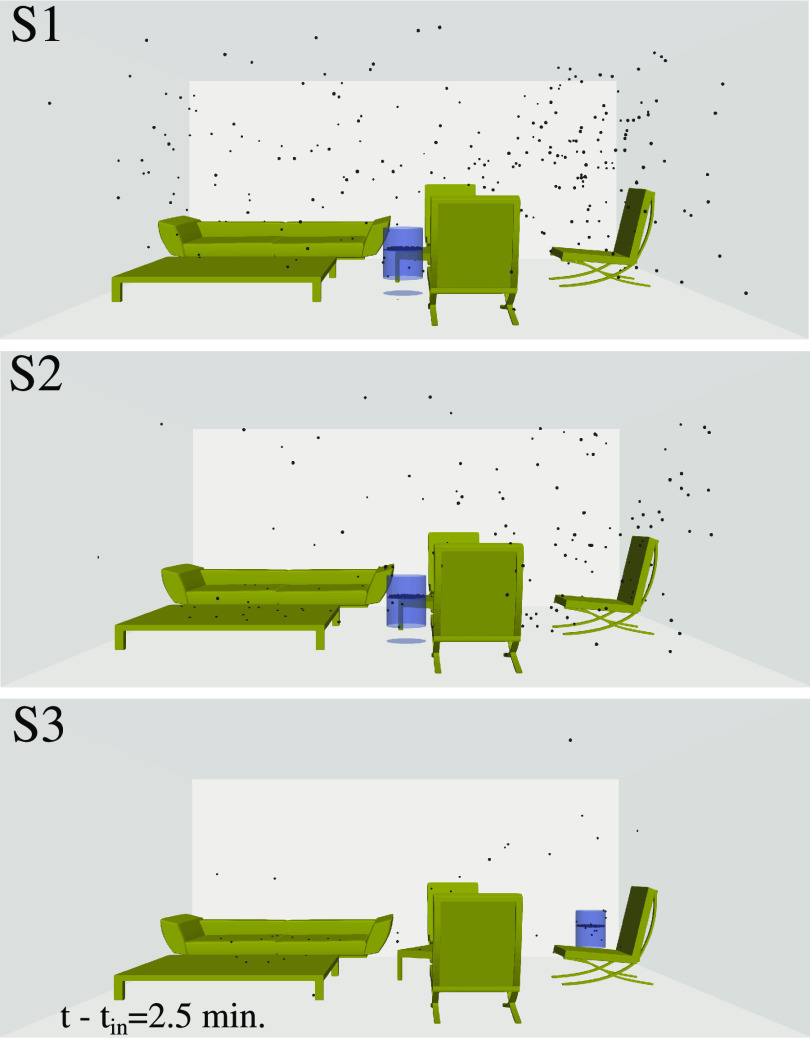
The local distribution of the airborne virus particles at $t-t_{in}=2.5\;\text{min}$ for the three different scenarios S1, S2, and S3 of Fig. 2.

Figure 5 (Multimedia view) shows the local distribution of the airborne virus particles at $t-t_{in}=1\;\text{min}$ under the three different scenarios: S1, S2, and S3 (see Fig. 2). A corner position for the air purifier is more effective under scenario S3. This is because the air intake is on top of the purifier device, and the air exhaust is at the bottom [see scenario S3 of Fig. 2(b-iii)]. The optimal local position of the purifier depends on the interior design and the shape and size of the furniture. The higher efficiency of the air purifier in scenario S3, i.e., cleaning more rapidly the airborne virus particles, is also shown for $t-t_{in}=2.5\;\text{min}$ in Fig. 6.

**FIG. 7.**
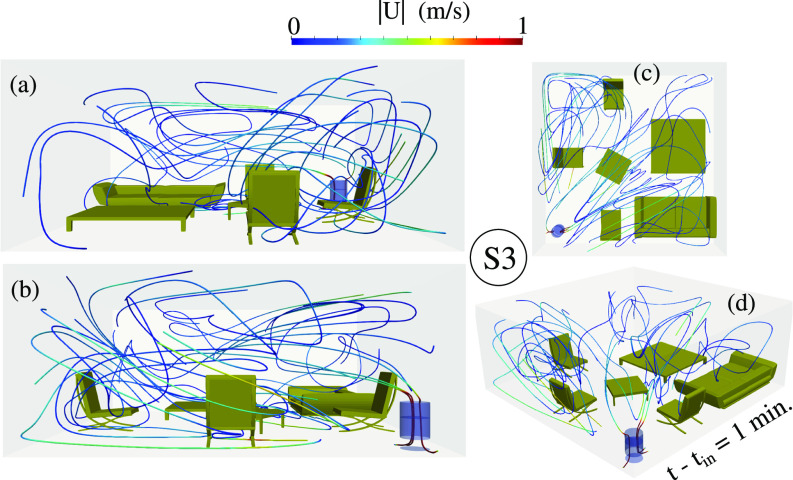
The air dynamics at $t-t_{in}=1\;\text{min}$ for the scenario S3 (Fig. 2) represented by streaklines from different angles: (a) front; (b) back; (c) top; and (d) 3D perspective. The streaklines are colored by the velocity magnitude per color bar.

**FIG. 8.**
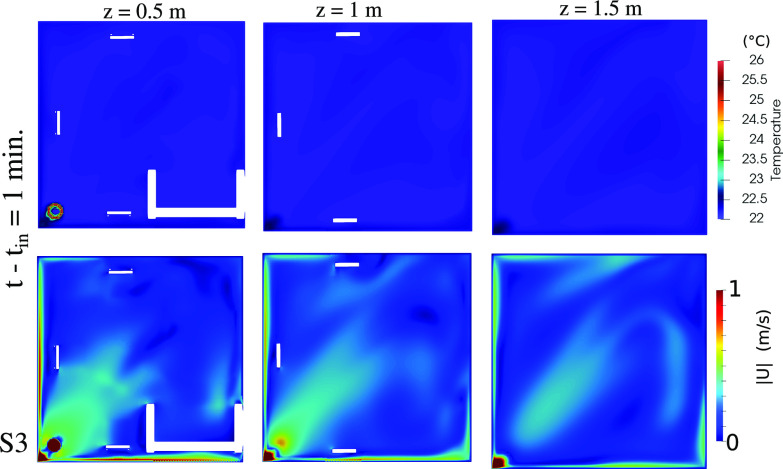
Indoor comfort level in terms of maximum local temperature (top row) and maximum local air speed (bottom row). Results at $t-t_{in}=1\;\text{min}$ for the scenario S3 (Fig. 2) and at different horizontal cross sections above the ground: (a) z = 0.5; (b) z = 1; and (c) z = 1.5 m.

We investigated the airflow dynamics inside the living room at $t-t_{in}=1\;\text{min}$ by examining the streaklines of Fig. 7 for the most efficient case (S3). The velocity magnitude colors the streaklines to illustrate the local comfort level in terms of the maximum speed that must not be higher than a maximum critical value. The latter usually should be less than or equal to 0.5 m/s, which may vary depending on the HVAC (heat, ventilation and air-conditioning) systems and the regulations set for different buildings. The comfort level in terms of local temperature and local airspeed is also analyzed for scenario S3, Fig. 8 at $t-t_{in}=1\;\text{min}$. The temperature is close to its initial value. The local air velocity away from the air purifier exhaust is lower than 0.5 m/s.

**FIG. 9.**
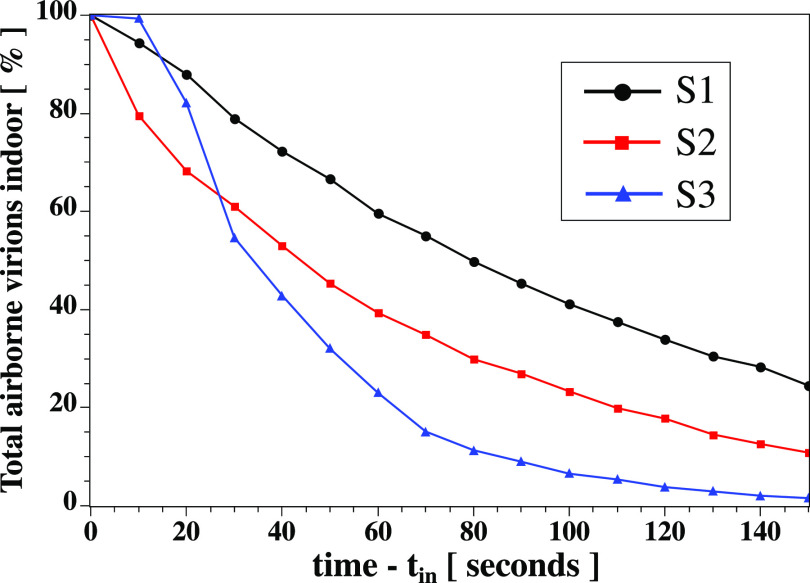
The total percentage of airborne virions suspended in the indoor space outside the air purifier device. Scenario S3 allows faster absorption of virus particles outside the air purifier device. This is because the particles are entrapped (or attracted) more quickly into the air purifier positioned at the corner of the indoor space (see Fig. 2).

Figure 9 illustrates the total percentage of airborne virions suspended in the air outside the air purifier device. Scenario S3 allows a more rapid treatment of the suspended virus particles outside the air purifier device. The airborne virus particles are attracted faster into the air purifier device positioned at the corner of the indoor space. At $t-t_{in}\leq 25\;s$, S2 outperforms S3. However, for $t-t_{in}>25\;s$, S3 outperforms both S1 and S2 by absorbing faster the virus particles into the air purifier.

It is worth noting that the precise effect of the purifier's position vs the furniture arrangement in the room, and the different types of furniture, e.g., taller bookcases, is a bespoke issue that requires optimization by performing a few simulations as there are several possible combinations. The optimization of the system's installation is beyond the scope of the paper.

## IV. A NEW CONCEPT OF AIR PURIFICATION: RESULTS AND DISCUSSION

**FIG. 10.**
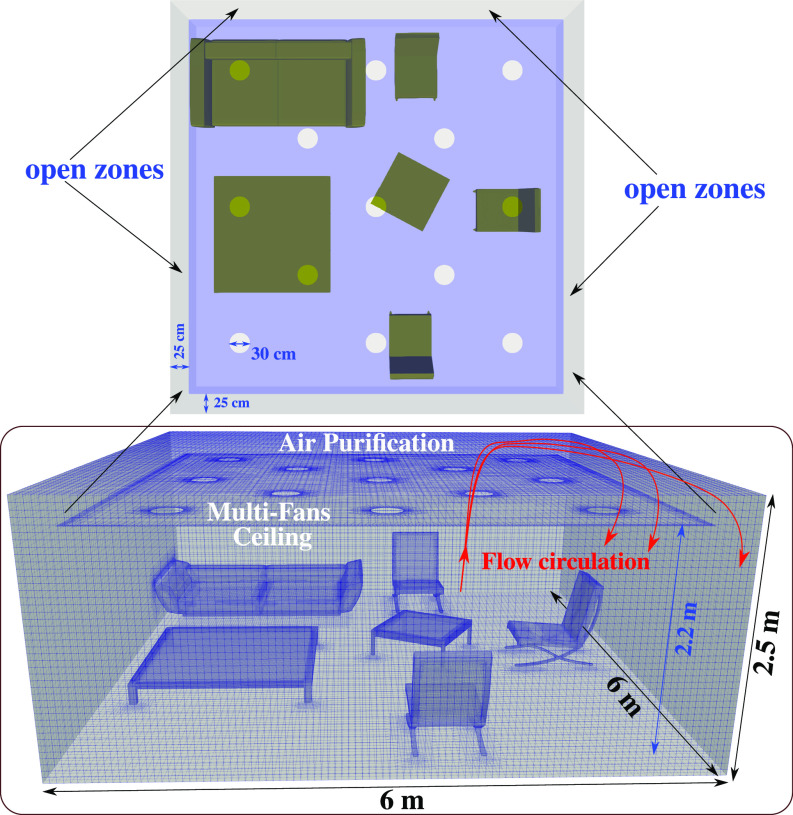
A new concept of air purification using 13 multi-fans (for rapid uniform air intake) installed close to the ceiling and operating at a flow rate of $100\;m^{3}/h$.

Given the above findings, we propose a new concept of air purification per Fig. 10 based on installing multiple in-ceiling fans 30 cm close to the upper wall operating at $T_{wf}=30\;{}^{\circ}C$. The aim is to entrap more rapidly the airborne viruses than the best performance obtained in scenario S3. The new concept employs 13 multi-fans (for a more rapid uniform air intake) installed close to the ceiling. They operate at a lower flow rate of $100\;m^{3}/h$ compared to that of the air purifier device employed in S3 (Fig. 2). The flow circulation occurs along the cross-section of the indoor space. The air exhausts are simple openings in the lateral directions close to the walls, e.g., open zones on the upper side of Fig. 10.

**FIG. 11.**
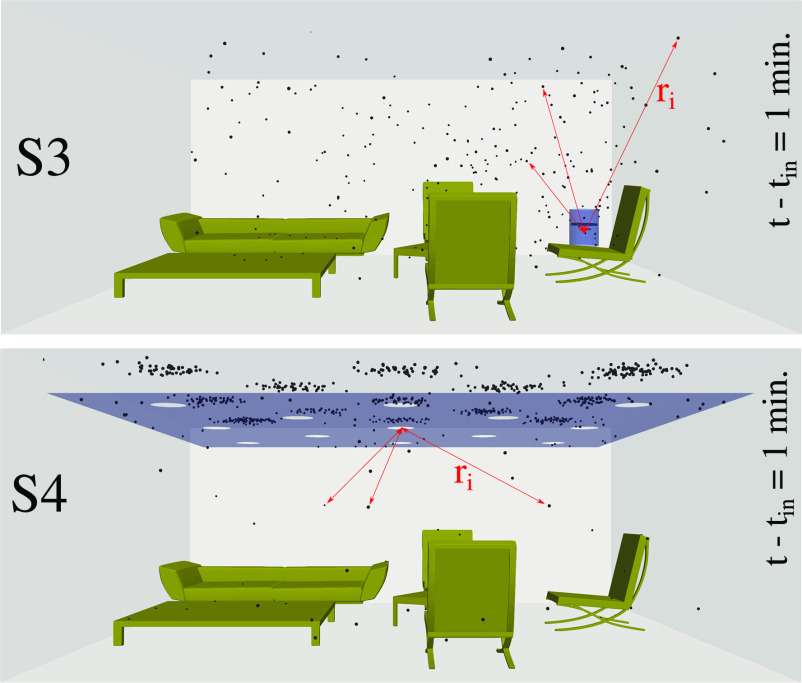
Distribution of airborne virus particles at $t-t_{in}=1\;\text{min}$ for cases S3 (Fig. 2) and S4 (Fig. 10). *r_i_* is defined as the radial position from the air purifier's center to the particles that are still freely suspended in the surrounding indoor air, and the subscript *i* denotes a particle's index. Scenario S4. Multimedia view: https://doi.org/10.1063/5.0064115.4
10.1063/5.0064115.4

The distribution of the airborne virus particles at $t-t_{in}=1\;\text{min}$ is shown in Fig. 11 (Multimedia view). We compare the cases S3 (Fig. 2) and S4 (Fig. 10). S4 provides faster entrapment of virions into the purification zone. S4 also directs the particles to the ceiling instead of letting them suspend in the environment, something that would increase the virus transmission.

**FIG. 12.**
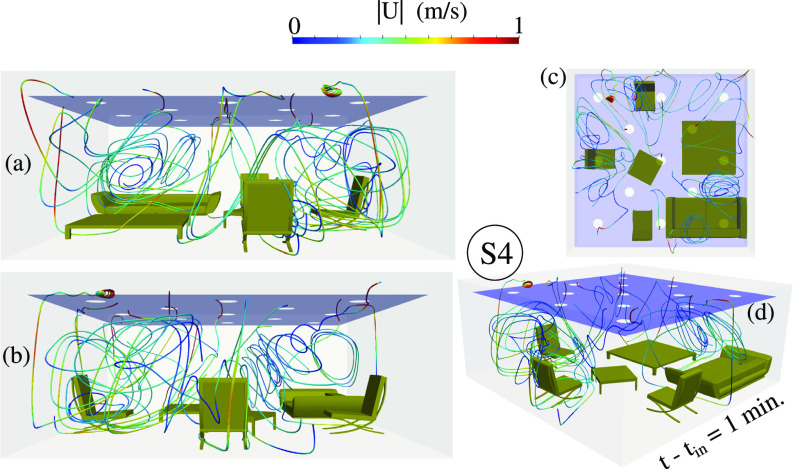
The local air dynamics at $t-t_{in}=1\;\text{min}$ for S4 (Fig. 10) represented by streaklines as seen from different angles: (a) front; (b) back; (c) top; and (d) 3D perspective. The streaklines are colored by the velocity magnitude (color bar).

**FIG. 13.**
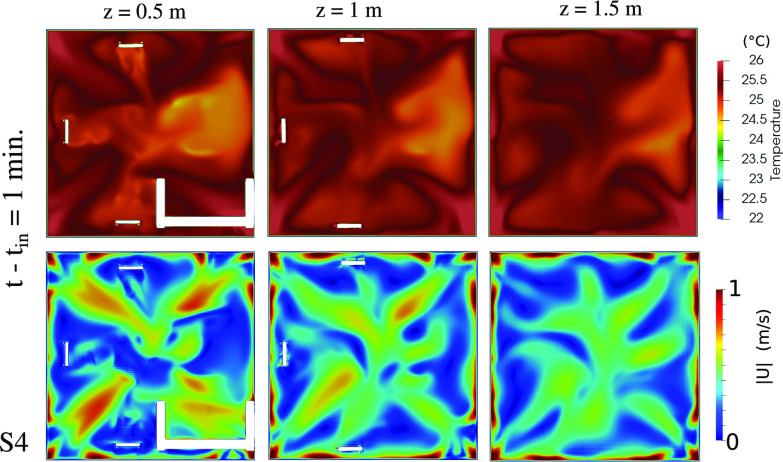
Indoor comfort level in terms of maximum local temperature (top row) and maximum local air speed (bottom row). Results at $t-t_{in}=1\;\text{min}$ for S4 of Fig. 10 and at different horizontal cross sections above the ground: (a) z = 0.5; (b) z = 1; (c) z = 1.5 m.

The airflow dynamics in the room for S4 is further analyzed at $t-t_{in}=1\;\text{min}$ using the streaklines (Fig. 12). S4 facilitates air circulation, evident by the vortical structures compared to the streaklines for S3 in Fig. 7. The comfort level in terms of local temperature and airspeed for S4 are shown in Fig. 13. The temperature is about 3 °C higher than the initial temperature. The local air velocity away from the air purifier is lower than about 0.7 m/s. The difference in the local temperature between S4 and S3 is due to the boundary condition imposed on the upper wall that includes multiple fans ( $T_{wf}=30\;{}^{\circ}C$).

**FIG. 14.**
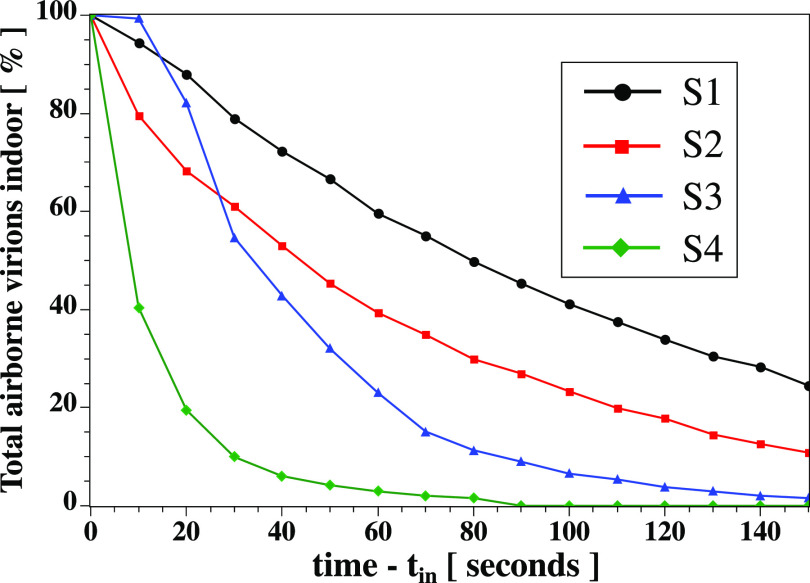
The total percentage of airborne virions that suspended in the air. S4 (Fig. 10) provides the best approach.

**FIG. 15.**
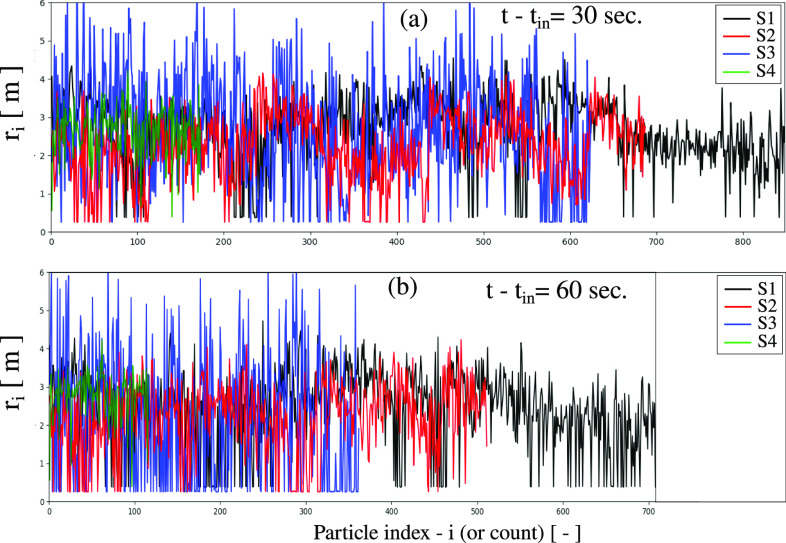
Quantitative analysis showing the local radial position *r_i_* of the freely-in-air suspended virus particles away from the air purifier's center, as defined in Fig. 11. (a) at $t-t_{in}=30\;s$ and (b) at $t-t_{in}=60\;s$.

For a better quantitative analysis of S4 performance, Fig. 14 shows the total percentage of airborne virions that are suspended in the air in the indoor space outside the air purification zone. S4 allows a faster treatment of the suspended virus particles compared to S3, S2, and S1. This due to the multiple fans installed in the ceiling close. The particles are absorbed fast toward the upper wall at z=2.5 m. The radial position of particles in the air is shown in Fig. 15 for all cases (S1 to S4). S4 outperforms S3, S2, and S1 at different times $t-t_{in}=30\;s$ and $t-t_{in}=60\;s$.

## V. CONCLUSIONS AND PERSPECTIVES

We investigated different scenarios to utilize indoor air purifiers to clean airborne viruses through computational fluid dynamics. We studied the local position of the purifier in a furnished living room and the direction of air from the fan installed in the purifier device. We found that the above can have significant effects on airborne virus transmission indoors.

Furthermore, we proposed a new indoor air circulation system that is more efficient in absorbing airborne virus particles than conventional approaches. The proposed concept uses in-ceiling multi-fans instead of placing small purifier equipment on the floor. This new concept allows faster airborne virus particles removal. We provide a few recommendations below:

• Regular DAP designs should absorb the infected air vertically from top to bottom and eject the clean air laterally from the bottom side of the device.
• Positioning the DAP device at the corner is more efficient as it reduces the time needed to bring suspended particles into the device.
• The DAP should be placed close to the ceiling to minimize the risk of transmission. It is also safer to absorb the particles from the bottom to top side of the ceiling.

Gravitational forces have no significant effect on airborne viruses (as small as 140 nm that can suspend in the indoor air environment for long periods). This suspension of tiny virus particles requires an optimal forced convection mechanism to be embedded into future air purifiers equipment (filter- or UV-LED based) to rapidly treat and efficiently attract the airborne virus into the device.

## Data Availability

The data that support the findings of this study are available from the corresponding author upon reasonable request.
